# Tripterygium drug-loaded liposome alleviates renal function by promoting vascularization and inhibiting fibrosis

**DOI:** 10.3389/fchem.2024.1427670

**Published:** 2024-07-01

**Authors:** Ziwei Chen, Jiajia Wang, Jianyu Zhou, Haifeng Yu, Lu Zheng, Yuan Chen, Xiaoqing Wan, Wei Zhang

**Affiliations:** ^1^ Department of Nephrology, Taizhou Central Hospital, Affiliated to Taizhou University, Taizhou, China; ^2^ Department of Traditional Chinese Medicine, Taizhou Hospital of Zhejiang Province, Taizhou, China; ^3^ Department of Ultrasound, Taizhou Central Hospital, Affiliated to Taizhou University, Taizhou, China

**Keywords:** kidney injury, acute kidney injury, renal fibrosis, liposome, mitochondria

## Abstract

**Introduction:** Tripterygium species have been traditionally used in Chinese medicine for treating various conditions. The aim of the study was to construct a drug-modified renal infarction targeting liposome (rTor-LIP) containing *Tripterygium* in order to improve the therapeutic effect on renal injury.

**Methods:** rTor-LIP was prepared using the extruder method containing *Tripterygium* solution. The preparation was characterized by transmission electron microscopy, Marvin laser particle size analyzer, and Western blotting. *In vitro* experiments were conducted to verify the biocompatibility of rTor-LIP, and *in vivo* experiments were conducted to verify the therapeutic effect of rTor- LIP on renal injury.

**Results and discussion:** The surface of rTor-LIP was regular and oval. In vitro results showed that after co-incubation with rTor-LIP, endothelial cells did not show significant apoptosis, and there were no significant abnormalities in the mitochondrial metabolism. The *in vivo* results showed that the morphology of endothelial cells in the rTor-LIP group was uniform and the cytoplasmic striations were clear, but the local striations had disappeared. Thus, rTor-LIP nano-targeted liposomes can effectively target hypoxic kidney tissue, providing a new idea for the treatment of renal infarction.

## 1 Introduction

The kidney is the most important excretory organ of the human body and has important physiological functions (Levey and James, 2017; Xu et al., 2021). Acute kidney injury (AKI) is a sudden decrease in kidney function that develops within 7 days, characterized by an increase in serum creatinine or a decrease in urine output, or both. Causes of AKI are classified as prerenal, intrinsic renal, or postrenal. Prerenal causes include sepsis, dehydration, and heart failure. Intrinsic renal causes include acute tubular necrosis and certain medications. Postrenal causes include kidney stones and bladder cancer. Diagnosis is based on the signs, symptoms, lab tests, and imaging (Bellomo et al., 2012; Zhang et al., 2022). AKI is observed in 10%–15% of hospitalized patients and over 50% of ICU patients. Complications include metabolic acidosis, high potassium levels, and uremia. AKI can lead to chronic kidney disease (Petejova and Martinek, 2014). Management of AKI involves treating the underlying cause and providing supportive care. Symptoms of AKI include fatigue, loss of appetite, and abnormal heart rhythms due to high potassium levels. Fluid balance can also be affected (Zhang et al., 2019).

Current treatments for AKI have limitations due to the complexity of disease mechanisms, regional variations in clinical practice, and a lack of effective therapies to promote kidney recovery (Kellum and Prowle, 2018; Zhang B. et al., 2021). Sepsis and AKI worsen the outcomes for critically ill patients, highlighting the urgent need for better therapeutics. Current treatments for AKI have limitations due to the challenges in translating successful animal model studies to human trials (Rahman et al., 2012). Despite extensive research on cellular and molecular mechanisms, clinical trials have not yielded conclusive results. The lack of epidemiological data in low-income countries further complicates the recognition and management of AKI (Claure-Del Granado et al., 2023). Treatment strategies often focus on addressing the underlying causes of metabolic acidosis, such as bicarbonate therapy for severe acidemia in AKI. For chronic metabolic acidosis, oral alkali therapy is commonly used to slow the progression of chronic kidney disease (Rangaswamy and Sud, 2018; Holt et al., 2023). However, this treatment may have side effects such as gastrointestinal intolerance and hypertension. Dietary interventions and protein restriction also exhibit promising results in correcting metabolic acidosis in patients with chronic kidney disease (Jury and Shaw, 2021). Novel compounds and delivery methods, such as renal artery infusion catheters, are being explored to improve the treatment of AKI.

Tripterygium species, including *Tripterygium wilfordii* Hook. F, have been traditionally used in Chinese medicine for treating various conditions such as rheumatoid arthritis, nephrotic syndrome, and lupus (Zhang Y. et al., 2021; Sun et al., 2022). Triptolide, an active component in *Tripterygium wilfordii* extracts, has shown multiple pharmacological activities, including anti-inflammatory, anti-tumor, and neuroprotective properties (Sakai, 2022). Recent studies have focused on understanding the pharmacodynamics, pharmacokinetics, and toxicology of triptolide to reduce its toxicity and improve clinical efficacy (Xu et al., 2016). Tripterygium and its extracts have been found effective in treating diabetic nephropathy by reducing urine protein and protecting renal function through anti-inflammatory, anti-oxidative, and anti-fibrotic mechanisms (Liu et al., 2019; Zhang et al., 2023). Despite potential adverse effects, Tripterygium and its extracts are considered alternative medicines for diabetic nephropathy when used cautiously (Zhang Y. et al., 2021). Further research is needed to determine safe and effective doses for specific target organs and diseases to maximize the benefits of Tripterygium in clinical practice. If Tripterygium is used in the treatment of AKI, we will try to break the limitation of the existing methods for the treatment of AKI and then use liposomes to prepare drugs loaded with Tripterygium to solve the problem of poor drug metabolism caused by poor retention of Tripterygium *in vivo* (Yu et al., 2018). This approach is a new and interesting combination and experiment (Brown, 2017; Zheng et al., 2023). This is not only a novel approach for the treatment of AKI but also a novel approach to expand the application of Tripterygium. Most importantly, it is an idea to improve the performance of traditional Chinese medicine with modern biomedical technology and expand the scope and clinical application of Tripterygium (Wong et al., 2012; Tang et al., 2013).

Therefore, to test this novel idea, a drug-modified renal infarction targeting liposome (rTor-LIP) containing Tripterygium was prepared. The performance of rTor-LIP and biocompatibility was checked, and the results showed that rTor-LIP drug loading showed excellent properties and has good biocompatibility. We then treated AKI mice with rTor-LIP and found that rTor-LIP promoted angiogenesis and reduced the degree of renal fibrosis. With the help of fluorescence technology, we found that rTor-LIP was effective in targeting renal tissue after AKI. *In vitro* co-culture experiments showed that rTor-LIP liposomes promoted viability of human umbilical vein endothelial cells (HUVECs). In addition, we found that rTor-LIP treatment effectively improved the glomerular filtration function after injury and reduced renal fibrosis. Our study confirmed the feasibility and effectiveness of using Tripterygium to prepare rTor-LIP for the treatment of AKI, providing new ideas for the treatment of AKI and the clinical application of Tripterygium. This study demonstrated the excellent performance of rTor-LIP in the treatment of AKI and the therapeutic effects at the cellular and tissue levels, which suggested that rTor-LIP has great potential in the treatment of AKI.

## 2 Materials and methods

### 2.1 Preparation of rTor-LIP

The extruder method for preparing liposome vesicles is an efficient and commonly used experimental method for extracting and purifying vesicles from cells. First, HK2 cells were cultivated in sufficient quantity and in good condition under sterile conditions; then, the cells were collected and washed multiple times with PBS buffer to remove the impurities from the culture medium. Next, the cleaned cells were resuspended with an appropriate amount of PBS to an appropriate concentration, and vesicles were prepared using an assembled 200 nm pore size cell extruder; the process was repeated 10 times by left and right pushing and pulling. During the extrusion process, the cells are subjected to shear forces when passing through polycarbonate membranes with different pore sizes, resulting in cell rupture and release of vesicles. The PBS used for resuspending cells in the rTor-LIP group contained 10 mg/mL of Tripterygium, allowing for the preparation of vesicular liposomes loaded with Tripterygium when passing through polycarbonate membranes. The liquid passing through the extruder, which contains vesicles released by cells, is collected.

### 2.2 Transmission electron microscopy

The rTOR-LIP samples were rinsed with 0.1 mol/L sodium cacodylate buffer and then fixed with 1% osmic acid (prepared from dimethyl arsenic acid buffer at 4°C) for 1.5 h, rinsed with buffer, dehydrated with gradient ethanol to anhydrous, replaced with acetone, and then soaked and embedded with Epon812. The droplets of the rTOR-LIP sample were dropped onto a 200 mesh copper mesh and were directly observed under transmission electron microscope with phosphotungstic acid staining.

### 2.3 Nanoparticle tracking analysis

The isolated rTOR-LIP samples were diluted with 1 × PBS buffer, and the particle size and concentration of rTOR-LIP were measured using the ZetaView PMX 110 instrument and ZetaView 8.04.02 software. In the experiments, NAT measurements were recorded and analyzed at 11 locations. The ZetaView system was calibrated with 110 nm polystyrene particles, and the temperature was maintained between 23°C and 27°C.

### 2.4 The *in vitro* release assay

The isolated rTOR-LIP samples were immersed in 1 × PBS buffer, and at the specified time point, the leachate was gently aspirated and subjected to concentration detection of Tripterygium by high-performance liquid chromatography. The sample was diluted to the appropriate range according to the predetermined concentration, and the diluted sample was injected into the injector of the high-performance liquid chromatograph. Appropriate parameters were set, including the flow rate, temperature, and detection wavelength. The sample was separated in the chromatographic column. During the separation process, the effective components of the torch flower roots passed through the chromatographic column along with the mobile phase and were captured by the detector at different time points.

### 2.5 Western blot

The samples isolated from the rTOR-LIP by the homogenate were separately mixed with the loading buffer and boiled in water at 100 °C for 10 min before use. The samples were separated by 12% sodium dodecyl sulfate polyacrylamide gel electrophoresis (SDS-PAGE), electrophoresis was performed at 80 V for 120 min, and transfer printing was carried out at 300 m A for 90 min. The proteins were transferred to the methanol-activated PVDF membrane. The cells were blocked with 5% skim milk powder TBST at room temperature for 2 h, and then CD9 and CD63 antibodies were added and incubated overnight at 4°C. The membranes were washed three times with TBST for 10 min each time; then the corresponding secondary antibodies were added and incubated for 1 h at room temperature, and the membranes were washed three times.

### 2.6 Cellular immunofluorescence

The HUVECs were seeded at a density of 1 × 10^5^/well in a special dish for laser confocal microscopy. The cells were seeded in steps, and 200 μL of the cell suspension was added to the dish. The groups were grouped as above, and each group had three duplicate samples. The solution was changed every other day, and the staining was performed after 9 days of culture. MitoTracker-Green mitochondrial fluorescent probe staining was used as viable cell staining. Before staining, the diluted MitoTracker Green staining solution was preheated at 37°C in an incubator in the dark for 10 min, the medium in the culture dish was discarded, and the culture dish was washed three times with PBS buffer. For staining, 1 mL diluted MitoTracker Green staining solution was added to the culture dish and incubated in the original incubator for 15–30 min. The staining solution was aspirated, and then 1 mL of Hoechst 33258 staining solution at 100 nM was added to coat the nuclei and incubated for 10 min. Staining was completed by flushing the well plates with PBS buffer three times. Attention should be paid to perform the laser confocal microscopy observation and image acquisition as soon as possible after washing is completed.

### 2.7 Acute kidney injury model

All experimental steps must strictly follow the animal ethics and experimental norms. We selected male C57BL/6 mice aged 8–12 weeks. Mice were placed on a thermal pad, and the skin and muscles on the left side were cut along the back to expose the renal pedicle. The renal ischemia time was recorded by clamping it with arterioles, and the kidney was observed to change from red to deep purple. After modeling, the mice were placed on a thermal pad to raise their body temperature and closely monitored.

### 2.8 Live-dead stain

The HUVECs were stained by adding 10 μL Calcein-AM staining solution and 20 μL PI staining solution to 5 mL PBS buffer; then 500 μL Calcein-AM/PI composite staining solution was added to the cells in each well, and the cells were incubated in the incubator in the dark for 30 min. Cell survival was evaluated by observing the live and dead staining under 490 nm and 535 nm wavelength excitation light, respectively.

### 2.9 Histology

After mice were sacrificed with a high concentration of carbon dioxide, the kidneys were quickly removed and fixed in 4% paraformaldehyde fixative for 12 h, with one exchange of solution, and then they were dehydrated, made transparent, and embedded in paraffin. The embedded paraffin blocks were sectioned into a thickness of 3.5 μm, stained with preconfigured HE staining solution, and finally scanned with 3DHISTECH.

### 2.10 Immunofluorescence of kidney

After mice were sacrificed with a high concentration of carbon dioxide, the kidneys were quickly removed and embedded with Sakura Tissue OCT. After embedding, the kidneys were immediately frozen in liquid nitrogen, equilibrated at −20°C for 2 h, and then cryosectioned with a thickness of 3.5 μm. Image acquisition was performed using a ZEISS 700 laser confocal microscope.

### 2.11 Statistical analysis

The data from these experiments were reported as mean ± standard deviation, and the significance was evaluated by two-way ANOVA (SPSS 22 software package, United States). *P* < 0.05 was considered statistically significant.

## 3 Results

### 3.1 The characteristics of rTor-LIP

**FIGURE 1:**
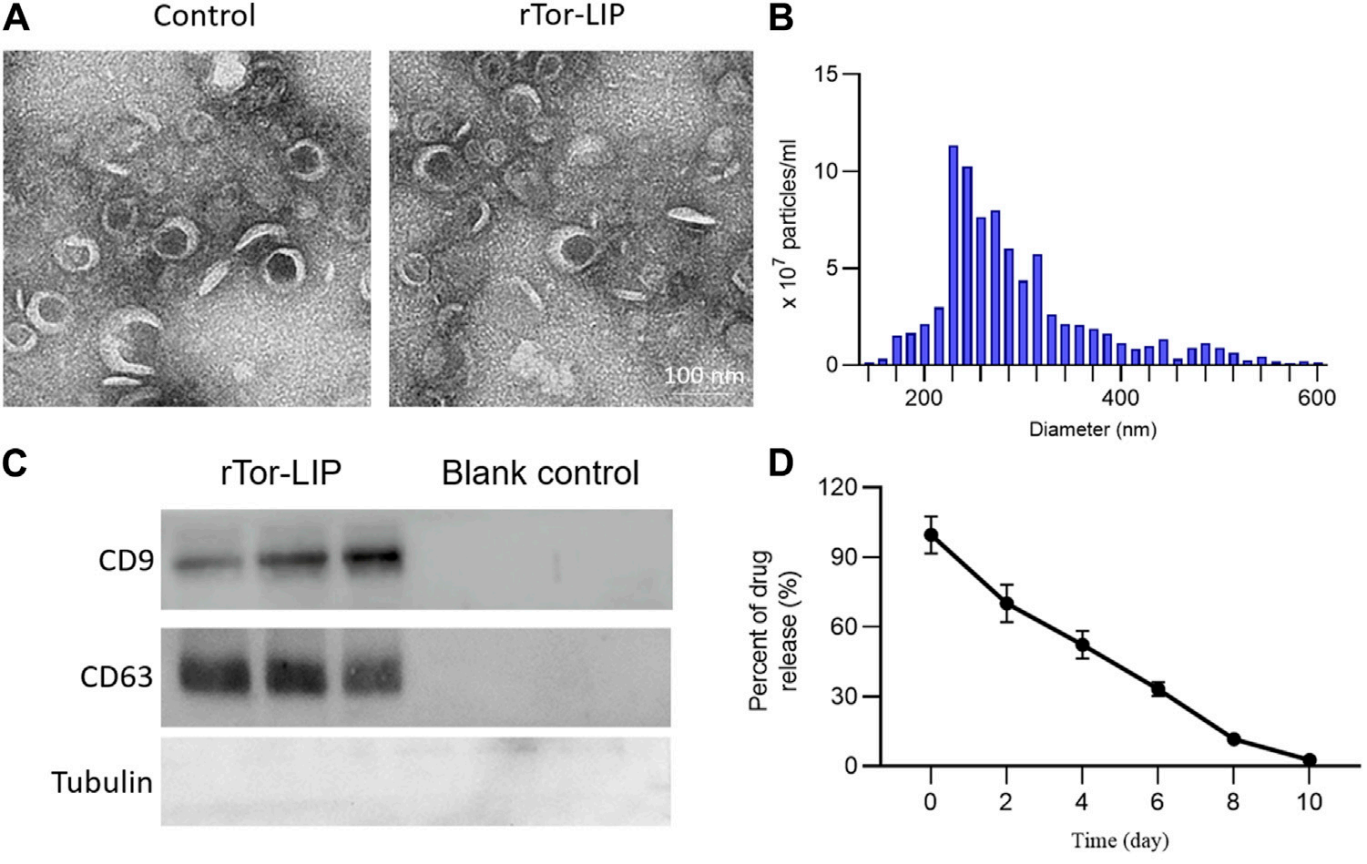
Characterization of rTor-LIP. **(A)** TEM imaging of the liposome without Tripterygium and rTor-LIP. **(B)** Particle diameter distributions of rTor-LIP. **(C)** Typical markers on rTor-LIP and the blank control. **(D)** Release behavior of the loaded Tripterygium from rTor-LIP.

First, we observed the morphology of the blank vesicles and vesicles loaded with *Tripterygium* using transmission electron microscopy. The results showed that rTor-LIP has very obvious membrane boundaries and presents an elliptical cup-shaped structure. The interior of rTor-LIP was observed to contain disorganized high electron density contents (Figure 1A). NTA analysis showed that the diameter range of rTor-LIP was distributed between 20 and 300 nm, with an average diameter of 266.17 ± 15.32 nm (Figure 1B). Next, the protein characteristics of rTor-LIP were analyzed by Western blot, and the results showed that the blank control group did not show any protein imprinting features, whereas rTor-LIP had obvious marker protein expression (Figure 1C). Characterization of Tripterygium release behavior by high-performance liquid chromatography revealed that rTor-LIP exhibits slow and controlled release characteristics. Tripterygium releases 65.82% ± 5.71%, 50.71% ± 3.99%, and 33.19% ± 2.74% at 2, 4, and 6 days, respectively (Figure 1D).

### 3.2 *In vitro* biocompatibility: no cytotoxicity

**FIGURE 2:**
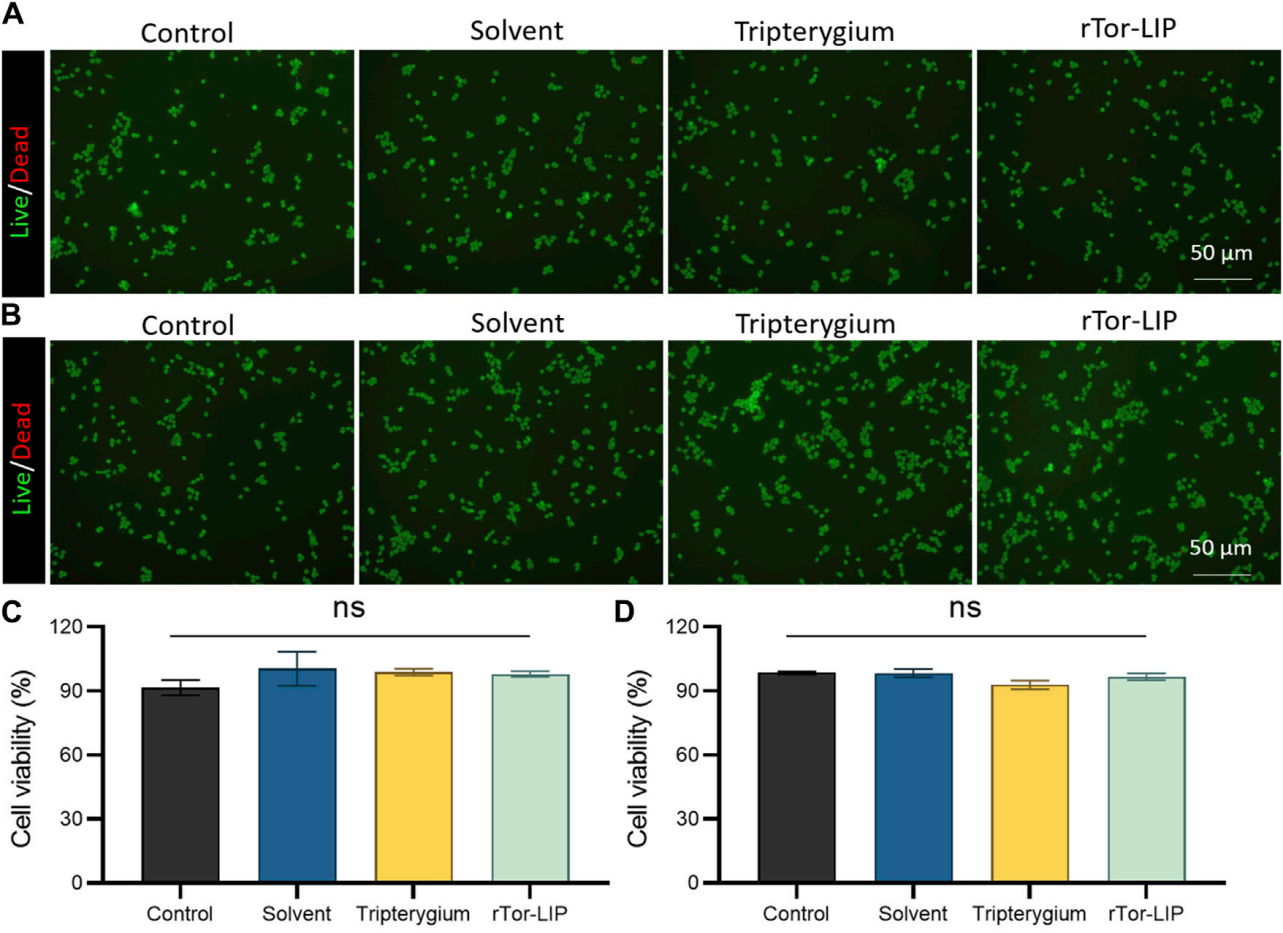
rTor-LIP had no adverse effects on cell survival. **(A)** Live and death staining of HUVECs co-cultured with solvent, Tripterygium, and rTor-LIP for 3 days. **(B)** Live and death staining of HUVECs co-cultured with solvent, Tripterygium, and rTor-LIP for 7 days. **(C)** Quantitative statistics of cell survival rate of HUVECs co-cultured with solvent, Tripterygium, and rTor-LIP for 3 days. **(D)** Quantitative statistics of cell survival rate of HUVECs co-cultured with solvent, Tripterygium, and rTor-LIP for 7 days. Data were presented as mean ± SEM, and multiple group comparisons were carried out using one-way ANOVA. Ns, no significance.

Biocompatibility is the primary prerequisite for the application of biomaterials, and we conducted a biosafety study of rTor-LIP regarding a co-culture experiment with HUVECs. The staining results of live/dead cells showed that on the third day of co-culture, the cell survival rates of the control group, solvent group, Tripterygium group, and rTor-LIP group were 90.36% ± 6.89%, 92.43% ± 6.19%, 91.38% ± 5.86%, and 91.27% ± 4.07%, respectively. There was no statistical difference among the four groups (Figures 2A, C). On the fifth day of co-culture, the cell survival rates of the control group, solvent group, Tripterygium group, and rTor-LIP group were 91.85% ± 4.18%, 92.03% ± 7.15%, 90.66% ± 6.08%, and 91.41% ± 5.87%, respectively. Similarly, there was no statistically significant difference in the results (Figures 2B, D). The above results revealed that on the third day and fifth day of co-cultivation of rTor-LIP and HUVECs, cell activity exceeded 90%, and there was no statistically significant difference among the groups. This indicated that rTor-LIP had minimal cytotoxicity to HUVECs, suggesting that rTor-LIP had good compatibility and no cytotoxicity, and could be widely used in various biological tissue therapy processes.

### 3.3 *In vivo* effect of rTor-LIP on endothelium

**FIGURE 3:**
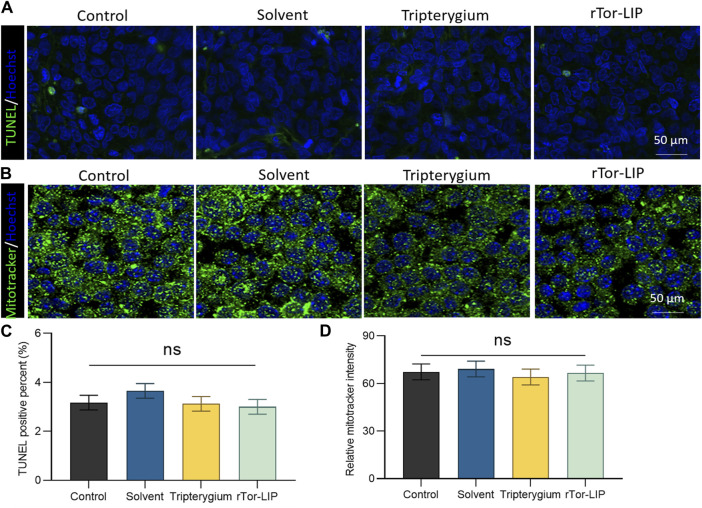
Biocompatibility of rTro-LIP to endothelial cells. **(A)** TUNEL fluorescence staining of HUVECs co-cultured with solvent, Tripterygium, and rTor-LIP for 3 days. **(B)** MitoTracker fluorescence staining of HUVECs co-cultured with solvent, Tripterygium, and rTor-LIP for 3 days. **(C)** Quantitative statistics of TUNEL positive cells in **(A)**. **(D)** Quantitative statistics of relative MitoTracker stain intensity in **(B)**. Data were shown as mean ± SEM, and statistical tests were performed by two-tailed *t*-test. Ns, no significance.

The biocompatibility of rTor-LIP is crucial for its application in the biomedical field. When rTor-LIP interacts with organisms, it can cause appropriate reactions; that is, it will not cause harmful biological reactions to the organism, and it will not cause adverse toxic reactions. First, after co-culturing rTor- LIP with HUVECs for 3 days, apoptosis of the cells was observed by TUNEL staining (Figure 3A). The results showed that the TUNEL positive rates in the control group, solvent group, *Tripterygium* group, and rTor-LIP group were 3.37% ± 0.21%, 3.69% ± 0.18%, 3.09% ± 0.16%, and 3.01% ± 0.20%, respectively, and no significant statistical differences were found among the groups (Figure 3C). Mitochondria are the “energy factories” of cells, providing a large amount of ATP to cells through the process of oxidative phosphorylation, ensuring that various life activities of cells can proceed normally. In addition, mitochondrial metabolism is not only related to energy production but also participates in important physiological processes such as signal transduction and apoptosis regulation within cells, which are crucial for maintaining cell homeostasis and adapting to environmental changes. Therefore, after co-culturing rTor-LIP with HUVECs for 3 days, MitoTracker staining was used to observe the mitochondrial status of the cells (Figure 3B). The results showed that the mitochondrial fluorescence staining intensities of the control group, solvent group, Tripterygium group, and rTor-LIP group were 63.28% ± 5.46%, 65.77% ± 5.29%, 61.57% ± 4.98%, and 64.58% ± 4.22%, respectively. No significant statistical differences were observed among the groups (Figure 3D). The above results indicate that rTor-LIP has good biocompatibility and no significant cytotoxic effects.

### 3.4 rTor-LIP improved angiogenesis and decreased apoptosis of mice kidney

**FIGURE 4:**
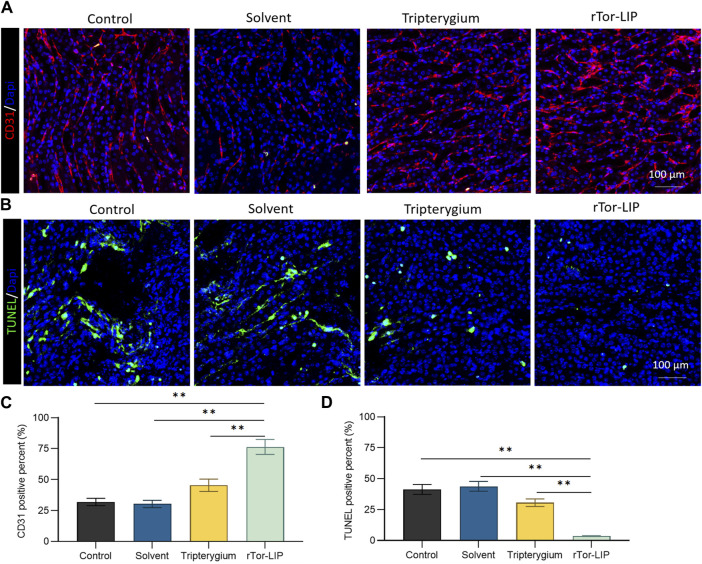
rTor-LIP improved angiogenesis and decreased apoptosis of mice kidney. **(A)** Representative images and positive CD31 cells staining on different groups of mice kidney tissue. **(B)** Representative images and positive TUNEL cells staining on different groups of mice kidney tissues. **(C)** Quantitative statistical diagram of angiogenesis in **(A)**. **(D)** Quantitative statistical diagram of apoptosis in **(B)**. Data were represented as the mean ± SD, **P < 0.01.

Vascularization is the foundation of normal kidney function, ensuring that the kidneys receive sufficient oxygen and nutrients while effectively eliminating metabolic waste. Vascularization is crucial for maintaining glomerular filtration function, as it ensures the filtration of harmful substances in the blood, thereby protecting the body’s health. The state of vascularization directly affects the progression and prognosis of kidney disease, and good vascularization can help improve the clinical outcomes of kidney disease. Therefore, we first performed immunofluorescence staining on endothelial cells using CD31 to evaluate the vascularization status of different groups after AKI (Figure 4A). The results showed that the CD31 positivity rate in the rTor-LIP group was 73.67% ± 5.26%, which was significantly higher than that in the control group, solvent group, and Tripterygium group, which were 24.51% ± 1.69%, 23.77% ± 1.96%, and 38.58% ± 3.21%, respectively (Figure 4C). Apoptosis of renal functional cells is an important pathological and physiological process after AKI, which has a direct impact on the recovery and degree of injury of the kidney. In further tests, immunofluorescence staining was performed on frozen sections of kidney tissue using TUNEL to evaluate the apoptosis of renal functional cells in different groups after AKI (Figure 4B). The results showed that the TUNEL positivity rates of the control group, solvent group, and Tripterygium group were 44.32% ± 3.78%, 47.10% ± 3.84%, and 28.17% ± 2.06%, respectively, which were significantly higher than the rTor-LIP group’s rate of 5.16% ± 0.63%, confirming the protective effect of rTor-LIP on renal function cells (Figure 4D).

### 3.5 rTor-LIP alleviated renal function after acute injury in mice

**FIGURE 5:**
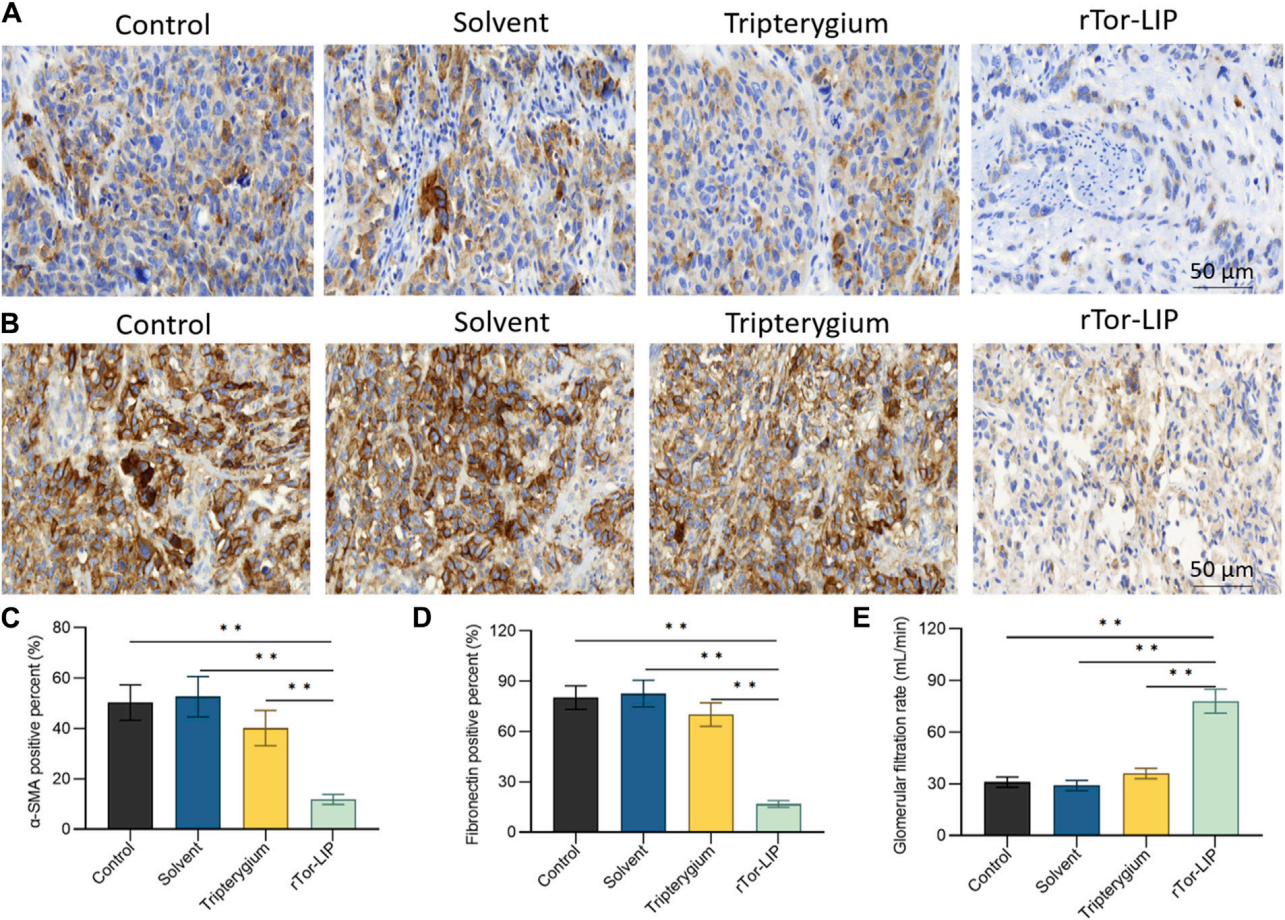
rTor-LIP alleviated renal function after acute injury in mice. **(A)** Representative images and analysis of α-SMA of mice kidney. **(B)** Representative images and analysis of fibronectin of mice kidney. **(C)** Quantitative statistical diagram of the α-SMA expression level in **(A)**. **(D)** Quantitative statistical diagram of the fibronectin expression level in **(B)**. **(E)** Glomerular filtration rate of mice in control, solvent, Tripterygium, and rTor-LIP groups. Data were represented as the mean ± SD, **P < 0.01.

To determine the role of rTor-LIP in improving the chronic changes and outcomes of AKI, we measured the severity of renal interstitial fibrosis in mice 28 days after AKI modeling using immunohistochemistry of alpha smooth muscle actin (α-SMA) (Figure 5A) and fibronectin (Figure 5B). Compared with the control group and solvent group, the fibrosis degree in renal specimens of the rTor-LIP group was significantly reduced by more than 70% (Figures 5C, D), indicating the potential of rTor-LIP to improve chronic fibrosis in AKI renal tissue. By analyzing the long-term renal function of the mouse AKI model, it was found that on the 28th day after injury, the glomerular filtration rate in the rTor-LIP group exceeded 60 mL/min, which is approximately twice that of the other groups (Figure 5E). These suggested that rTor-LIP provided good protection for renal function after AKI. The above results indicated that the application of rTor-LIP could alleviate chronic fibrosis and protect long-term renal function in mice with AKI.

## 4 Discussion

Acute kidney injury (AKI) is a common and serious clinical disease, which is often accompanied by a variety of serious complications (Bhargava and Schnellmann, 2017; Maekawa et al., 2019; Volarevic et al., 2019; Yoo et al., 2020). In nearly a decade, the incidence of AKI significantly increased. Each year, more than 10 million people worldwide suffer from AKI, and among them, more than 1.5 million people died of AKI and its complications (Kher and Kher, 2020). A meta-analysis showed that the incidence of AKI in adult inpatients worldwide was 21.6% (Susantitaphong et al., 2013). The incidence and mortality of AKI in intensive care unit patients were higher than those in general wards, and the long-term prognosis of acute kidney injury was poor (Hoste et al., 2015). At present, most epidemiological studies in high-income countries are multi-center and large-sample studies, and most of the data come from large databases, but prospective studies are rare (Tang et al., 2019; Weng et al., 2021).

This study explored the therapeutic effect of rTor-LIP on AKI. Although previous studies have explored ways to avoid and reduce the risk of AKI caused by disease factors and treatment methods, and summarized many factors leading to AKI in order to prevent the occurrence of AKI, effective treatment strategies and methods are still limited for facing the actual occurrence of AKI (Moore et al., 2018; Vijayan, 2021). We found that rTor-LIP prepared using the extrusion method had positive therapeutic effect on AKI. After verification by transmission electron microscopy and nanoparticle tracking analysis, we found that the rTor-LIP prepared by the extrusion method had excellent properties. The particle size of rTor-LIP was in the nanometer level, the boundary was clear, and high electron density contents could be observed. Regarding pharmacokinetic aspects, Western blot analysis showed that rTor-LIP has obvious signs of protein expression, and the result of the high-performance liquid chromatography (HPLC) shows rTor-LIP can be effectively used for controlled drug release, which solved the problem of the drug being stranded. Biocompatibility is the primary prerequisite for biomaterials application, so another important result of our study is that RTOR-LIP has good biocompatibility. Due to the toxicity of *Tripterygium*, *Tripterygium* should be used with caution in traditional Chinese medicine. Therefore, the biocompatibility of rTor-LIP was carefully validated. RTor-LIP did not show any adverse effect on the survival of HUVECs. We followed up the cell viability of HUVECs co-cultured with tripterygium for several days. It was found that the cell viability was more than 90%, the apoptosis index was normal, and there was no statistically significant difference among the groups. This indicates that rTor-LIP has minimal cytotoxicity to HUVECs, suggesting that rTor-LIP is biocompatible and non-cytotoxic, and can be widely used in various biological tissue therapeutic processes. Mitochondria are the “energy factory” of cells, which provide a large amount of ATP for cells through oxidative phosphorylation to ensure the normal operation of various life activities of cells. In addition, mitochondrial metabolism is not only related to energy production but also participates in important physiological processes such as intracellular signal transduction and apoptosis regulation, which are crucial for maintaining cellular homeostasis and adapting to environmental changes (Forbes and Thorburn, 2018). Therefore, mitochondrial health is also one of the indicators of our rTor-LIP. The intensity of mitochondrial fluorescent staining was normal in cells applied to rTor-LIP. Moreover, the biological activity of rTor-LIP is crucial. Vascularization is the basis for maintaining the health of the kidneys. The renal vascular network ensures that the kidney receives sufficient oxygen and nutrition, and it can effectively transport metabolic waste in the kidney (Sampaio, 2000). Vascularization is essential for the maintenance of glomerular filtration function because it ensures the filtration of harmful substances from the blood, thereby protecting the health of the body (Karim et al., 2020; Ren et al., 2020; Wang et al., 2022; Wu et al., 2022). Therefore, the status of vascularization directly affects the progression and prognosis of renal disease, and good vascularization helps to improve the clinical outcome of renal disease. After tracking the expression of CD31, we found that rTor-LIP promoted renal angiogenesis in AKI animals, and it also reduced the degree of apoptosis in renal functional cells as found by the TUNNEL assay. It is well known that fibrosis can lead to organ structural damage and dysfunction, even failure, which threatens human health and life (Liu et al., 2022; Wang et al., 2024). Fibrosis is mainly characterized by the increase of fibrous connective tissue in organ tissues (Rane et al., 2019; Bai and Liu, 2021; Li et al., 2022). Therefore, we measured the severity of renal interstitial fibrosis in mice with AKI by immunohistochemistry for α-smooth muscle actin (α-SMA) and fibronectin. Results showed that treatment with topical rTor-LIP had a good protective effect on renal function after AKI. Application of rTor-LIP could reduce chronic fibrosis and protect long-term renal function in mice with AKI. All these evidences suggest that rTor-LIP exerts positive effects on AKI at both the cellular and tissue levels. Therefore, we believe that rTor-LIP qualifies as a new idea and a new direction for AKI treatment. At the same time, our study also has some shortcomings. The toxicity of *Tripterygium* itself may have a low or high effect on the effect extent of rTor-LIP, and fully determining the dosage of Tripterygium in rTor-LIP requires a lot of additional time and effort. Therefore, long-term studies may be needed to determine an optimized and stable manufacturing process, especially with regard to the effect extent. Traditional Chinese medicine (TCM) is a treasure house with thousands of years of experience. Among them, thousands of natural medicines have unique therapeutic effects, which have great potential and application value to the research in modern medicine. In addition, our study shows that rTor-LIP has a significant therapeutic effect on AKI. In the future study, we may explore the liposomal formulation of other components for AKI treatment and further optimize the method for mass production of rTor-LIP. The abovementioned findings on the excellent performance of rTor-LIP in AKI treatment, as well as the positive treatment effect in both the cell and tissue levels, prove that rTor-LIP has great application potential in AKI treatment.

## 5 Conclusion

Our study shows that rTor-LIP has a significant therapeutic effect on AKI. In future studies, we may explore the liposomal formulation of other components for AKI treatment and further optimize the method for mass production of rTor-LIP. In view of the excellent performance of rTor-LIP in the treatment of AKI and the obvious therapeutic effect of rTor-LIP at the cellular and tissue levels, it has been proven that rTOR-LIP has great potential in the treatment of AKI.

## Data Availability

The raw data supporting the conclusion of this article will be made available by the authors, without undue reservation.
