# Supplementary figures and images for: Tripterygium drug-loaded liposome alleviates renal function by promoting vascularization and inhibiting fibrosis
Source: Front Chem. 2024 Jul 1;12:1427670. doi: 10.3389/fchem.2024.1427670 (PMC11246911; doi:10.3389/fchem.2024.1427670)

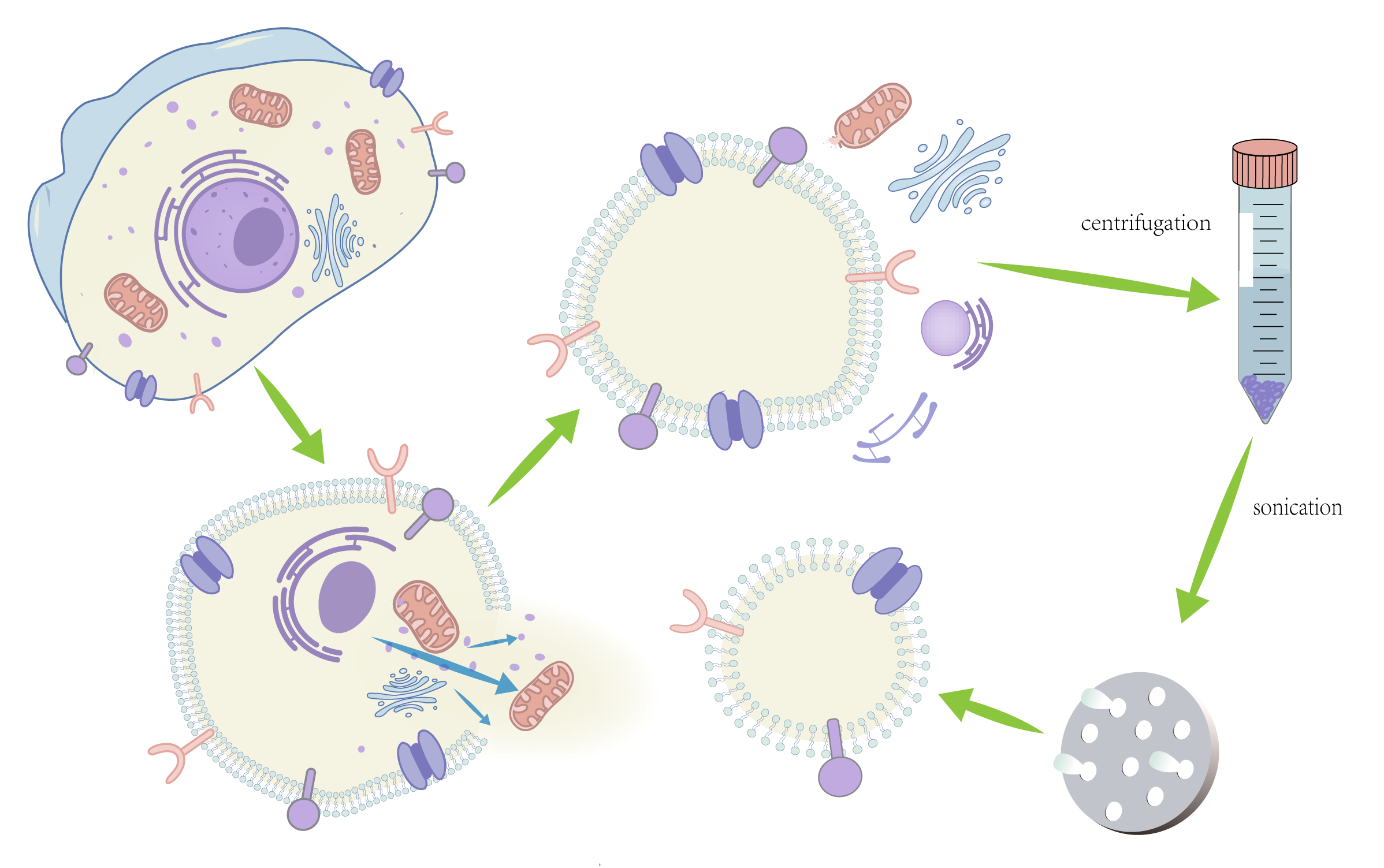

Supplement: Supplementary file 1 [file Image1.TIF]
